# Cross-cultural adaptation and psychometric validation of the simplified Oral Frailty Index (OFI-8) among Indonesian older adults

**DOI:** 10.3389/froh.2026.1853252

**Published:** 2026-08-07

**Authors:** Astari Larasati, Lindawati S. Kusdhany, Melissa Adiatman, Muslita Indrasari, Diah Ayu Maharani

**Affiliations:** 1 Faculty of Dentistry, Universitas Indonesia, Jakarta, Indonesia; 2 Department of Prosthodontics, Faculty of Dentistry, Universitas Indonesia, Jakarta, Indonesia; 3 Department of Dental Public Health and Preventive Dentistry, Faculty of Dentistry, Universitas Indonesia, Jakarta, Indonesia

**Keywords:** Indonesia, OFI-8, older adults, oral frailty, oral function, validity and reliability

## Abstract

**Background:**

Oral frailty refers to the manifestation of decreased oral function, such as poor oral health, impaired articulation, episodes of choking, and an increase in the amount of food that cannot be chewed. Recent studies have reported that oral frailty is a risk factor for physical frailty, sarcopenia, cognitive decline, and lower quality of life in older adults; however, it is challenging to detect deterioration in oral function since it requires specialist training and equipment. Alternatively, the Oral Frailty Index-8 (OFI-8) is a simple screening tool that can be implemented for community-level assessments, but this tool is not yet available in Indonesian.

**Aim and objectives:**

This study aims to cross-culturally adapt and assess the psychometric properties of the OFI-8 instrument among Indonesian older adults.

**Methods:**

A cross-sectional observational study was conducted involving 145 older adults aged 60 and above at Taman Sari Public Health Service, West Jakarta. This study was conducted in two stages: cross-cultural adaption of the OFI-8 (stage I) and assessment of psychometric properties of the OFI-8-ID (validity and reliability test, stage II).

**Results:**

Item-level Content Validity Index (I-CVI) values ranged from 0.83 to 1 and the Scale-level Content Validity Index (S-CVI/Ave) was 0.93, indicating good content validity for the OFI-8-ID. A strong positive correlation between the OFI-8-ID total score and total number of missing teeth was noted (*r* = 0.526). The intraclass correlation coefficient was 0.92 in 35 respondents, with values ranging between 0.68 and 1 for eight questions of the OFI-8-ID.

**Conclusion:**

The OFI-8-ID demonstrated good psychometric qualities and is a valid and reliable tool for measuring oral frailty in Indonesian older adults.

## 1 Introduction

Aging presents both challenges and opportunities. According to 2020 data from the Population Reference Bureau (PRB), Indonesia has the highest number of older adults with 15.16 million, or 5.6% of the population, followed by Pakistan with 9.31 million and South Korea with 7.83 million. Outside Southeast Asia, Japan has the largest proportion of older adults in the world, with 28.2% of the population (35.58 millions) (1). Although longevity is improving, it is not always accompanied by good health (2). From a generational standpoint, older adults will significantly impact healthcare demands, as this age group experiences deterioration in overall welfare and quality of life and is typically more fragile than younger individuals (2).

Oral health is an important component of health and social wellbeing, with significant impacts on overall health and quality of life, particularly for older adults. It encompasses not only the number of teeth present and the degree of oral hygiene but also oral functions such as mastication, tongue and lip motor functions, and salivary production (3, 4). Oral function refers to a precise motor skill responsible for performing activities such as speaking, chewing, swallowing, and coordinating tongue and lip movements, as well as saliva secretion (5, 6). Oral function is currently assessed using instruments that examine oral hygiene, oral dryness, number of teeth present, masticatory ability, tongue and lip motor function, tongue pressure, and difficulties in eating and swallowing.

Oral health is closely related to appetite, malnutrition, and social activities. Recently conducted studies have revealed that older adults have oral health issues, including decreased of oral function manifested as missing teeth, poor oral hygiene, and difficulties in eating and swallowing (7–9). These conditions are referred to as oral frailty and can result in diminished physical health, cognitive impairment, compromised social life, and a lower quality of life. The prevalence of oral frailty was investigated in a study involving 2,478 elderly individuals in Kusatsu City, which reported oral frailty rates of 22.3% in men and 22.7% in women (10). A previous cohort study in Japan revealed that fewer remaining teeth, edentulism, chewing difficulty, and xerostomia were significantly associated with an increased risk of dementia onset. Therefore, the risk of dementia onset may be reduced by maintaining good oral hygiene (11, 12). Difficulties in eating and swallowing may lead to dysphagia and masticatory dysfunction, which are directly linked to malnutrition and ultimately lead to poor general health. Prior research has found that dysphagia is an independent risk factor for sarcopenia, and decreased masticatory ability is significantly correlated with sarcopenia. In particular, poor oral function has been identified as a risk factor for frailty, sarcopenia, and reduced health-related quality of life (13–15). Thus, early assessment of oral function is crucial to mitigate the decline in overall health at its earliest stages.

The decline of oral function can be challenging to identify and may become irreversible by the time it is apparent. Oral function assessments are not routinely performed in many settings because they require specialized equipment and trained personnel. Several instruments have been developed to assess oral frailty, including the Oral Frailty five-item checklist (OF-5), the Screening Tool for Oral Frailty (SOFT), and the Oral and Maxillofacial Frailty Index (OMFI) (7, 16, 17). OF-5 is a shortened version of the oral frailty screening instrument developed in Japan. The original OF-5 study evaluated chewing difficulty, swallowing difficulty, and dry mouth using subjective questionnaires, whereas objective assessments were employed for tooth count and articulatory oral motor skills (18, 19). Concordance between subjective and objective evaluations was reported to be high (18). In 2024, the Japanese Geriatrics Society, the Japanese Geriatric Dentistry Society, and the Japanese Society for Sarcopenia and Frailty issued a consensus statement. This statement replaced the objective assessment of articulatory oral motor skills with the following self-reported question: “Have you had difficulty with clear pronunciation recently?” It also recommended allowing self-reported tooth counts (>20 teeth). However, further research is required to determine whether self-reported OF-5 assessments can accurately predict physical frailty outcomes (18). SOFT is a relatively new instrument for identifying older adults at risk of oral frailty, particularly in community and public health settings where comprehensive oral function assessment may not be feasible (16). This instrument was developed in China and consists of six items: number of teeth, difficulty swallowing, difficulty chewing, difficulty with articulatory motor function, dry mouth, and oral pain. Each item is answered as a yes-or-no question (16). The OMFI is another example of an instrument that can be used for oral frailty screening in older adults. This instrument was developed in Korea and consists of five items: difficulty chewing, the necessity of water when eating dry food, difficulties in jaw or tongue movements, difficulties in speaking or pronunciation, and difficulties in facial expression (17). However, only preliminary studies have used these two instruments, and the OMFI survey was conducted over telephone interviews. The validity of the instruments has not yet been evaluated. Moreover, some of these instruments such as OF-5 incorporate clinical examinations and objective functional assessments, meaning that their implementation may require specialized equipment, trained personnel, or additional clinical resources.

In 2018, Tanaka proposed the Oral Frailty Index-8 (OFI-8) to help screen older adults at risk of oral frailty in community settings (7, 19–21). The OFI-8 captures multiple dimensions of oral frailty, including chewing difficulty, swallowing function, and oral dryness. Moreover, it also evaluates social participation and dental habits (denture use and dental visit frequency), which are not included in other oral frailty screening instruments (7, 18). This instrument has also been used widely in many studies on oral frailty. Because of its simplicity, ease of administration, and suitability for community-based screening, the OFI-8 may be particularly useful for identifying older adults at risk of oral frailty in large populations (7, 18). However, this instrument is not yet available in an Indonesian version. Therefore, the present study focused on the cross-cultural adaptation and psychometric validation of the OFI-8 for use among Indonesian older adults.

## 2 Material and methods

### 2.1 Design, setting, and participant

This cross-sectional study was conducted in two stages. Stage 1 involved translation and cross-cultural adaptation of the OFI-8, and Stage 2 involved evaluating its construct validity and reliability. Inclusion criteria for were as follows: (i) community-dwelling older adults aged ≥60 years, (ii) ability to communicate independently in Bahasa Indonesia, and (iii) consent to participate. Recruitment consisted of consecutive convenience sampling of eligible respondents who attended a biweekly older adults program at the Taman Sari Community Health Center in Jakarta Barat between April and July 2025. Sample size was calculated using G*Power 3.1.9.6 with a 0.5 effect size, alpha level of 0.05%, and power of 95%. The minimum required sample size was 138 participants. This study was reviewed and approved by the Dental Research Ethics Committee, Faculty of Dentistry, Universitas Indonesia (No. 131/Ethical Approval/FKGUI/I/2025).

### 2.2 Methods

#### 2.2.1 Stage 1 (translation and cross-cultural adaptation)

This study followed the guidelines and recommendations of Beaton et al. for cross-cultural adaptation (22). The English version of the OFI-8 consists of eight dichotomous screening questions representing oral functions such as tooth loss, masticatory performance, maximum tongue pressure, articulatory oral motor skills, subjective difficulty in eating tough food, oral health behavior, and social participation. In the original OFI-8 scoring system, tooth loss, subjective chewing difficulty, and subjective swallowing difficulty are scored higher because they were identified as primary indicators of oral frailty (7) (Table 1). The total OFI-8 score ranges from 0 to 11 points, with higher scores indicating higher risk of oral frailty. Screening criteria are defined by the sum of the following scores: low risk 0–2 points, risk 3 points, and high risk more than 4 points.

**Table 1:** Items of the oral frailty index-8.

No	Items of OFI-8	Yes	No
Q1	Do you have any difficulties eating tough food compared to 6 months ago?	+2 points	
Q2	Have you choked on your tea or soup recently?	+2 points	
Q3	Do you use dentures?^a^	+2 points	
Q4	Do you often have a dry mouth?	+1 points	
Q5	Do you go out less frequently than you did last year?	+1 points	
Q6	Can you eat hard foods like squid jerky or pickled radish?		+1 points
Q7	How many times do you brush your teeth in a day? (two or more times/day)		+1 points
Q8	Do you visit a dental clinic at least annually?		+1 points

a If you have lost a tooth, it is important to treat it with a denture so that you can eat hard food.

Two translators—one with a medical background and one without—worked independently in a blinded forward-translation process. A committee approach method was then adopted to synthesize the two translations into a single version. This approach consisted of a meeting with the researchers to discuss translation differences and reach a consensus on the most appropriate translation for each item. Some modifications were made in this phase. During the meeting, one of the researchers moderated the discussions and documented the decisions made.

Back-translation was performed by two independent native translators in a blinded manner. Neither translator had access to the English version of OFI-8 and was not informed about its construct. Two versions of the OFI-8 in English were generated. A discussion was conducted by the expert committee—consisting of researchers, a gerodontology specialist, and a prosthodontist—to determine whether minor discrepancies exist between the two versions. In addition, this phase aimed to determine whether any changes in meaning had occurred between the items in the back-translation and the items in the original instrument. All versions of the OFI-8 (the English version, the translated version, and the back-translated version) were compared by the expert committee to identify possible ambiguities and discrepancies, and decide upon the most appropriate translation.

#### 2.2.2 Stage 2 (validity and reliability testing)

A cross-sectional study with 145 respondents was conducted to assess the validity and reliability of the OFI-8-ID. Content validity was evaluated following the forward- and backward-translation processes by calculating the Content Validity Index (CVI). Three experts in clinical prosthodontic, geriatric dentistry, and questionnaire adaptation independently assessed the relevance of each item in the Indonesian version of the OFI-8 using a four-point Likert scale (1 = not relevant; 2 = somewhat relevant; 3 = quite relevant; 4 = highly relevant). The item-level Content Validity Index (I-CVI) was calculated as the proportion of experts assigning a relevance rating of 3 or 4 to each item. The scale-level Content Validity Index, based on the average method (S-CVI/Ave), was calculated as the mean of the I-CVI values across all items. The CVI should ideally be 1.00 if the expert panel consists of three to five members (23, 24).

To ensure that the newly adapted Indonesian version of the OFI-8 was clear, unambiguous, and easily understood by the target population, a quantitative face validity assessment was conducted. The OFI-8 was pretested with 33 respondents representing the target population. Respondents were asked to independently evaluate each of the eight items on a four-point Likert scale assessing clarity and comprehensibility, where 1 = not clear/comprehensible; 2 = item needs major revisions; 3 = item needs minor revisions; and 4 = very clear/comprehensible (25). The qualitative ordinal responses were subsequently dichotomized to calculate the Face Validity Index (FVI). Ratings of 1 and 2 were assigned a score of 0 (non-agreement), whereas ratings of 3 and 4 were assigned a score of 1 (agreement). The item-level Face Validity Index (I-FVI) was calculated as the proportion of respondents giving a rating of 3 or 4 divided by the total number of respondents. The scale-level Face Validity Index, Averaging Method (S-FVI/Ave), was calculated by computing the arithmetic mean of all individual I-FVI values across the eight items (25). Qualitatively, the face validity of the OFI-8 was also pretested by asking the respondents for feedback regarding the clarity, comprehensibility, and relevance of the items.

The validity of each item was assessed using Pearson’s product-moment correlation between the item scores and total scores. The instrument was considered valid if the correlation value (Pearson’s correlation) was positive and the *p-*value (two-tailed) was less than 0.05, or if the *r*-count value exceeded the *r-*table value (the *r-*table value for a sample size of 145 is 0.163).

Concurrent validity was also tested by correlating the total score of the OFI-8-ID with the total number of missing teeth as a clinical indicator. High concurrent validity was indicated by a strong positive correlation between these measures.

Construct validity was further evaluated using exploratory factor analysis (EFA) to examine the underlying factor structure of the Indonesian version of the OFI-8. Prior to factor extraction, data eligibility for factor analysis was tested using the Kaiser–Meyer–Olkin (KMO) measure of sample adequacy and Bartlett's test of sphericity. A KMO value ≥0.50 and Bartlett's test being statistically significant (*p* < 0.05) were considered the minimum thresholds for proceeding with EFA. Principal component analysis was used as an exploratory approach to examine item clustering.

Internal consistency of the OFI-8-ID was assessed using Cronbach's alpha for the total scale. Item–total correlations were also calculated to evaluate how well each item correlates with the total score (excluding that item). A commonly accepted interpretation is ≥0.50 = Strong; 0.30–0.49 = Acceptable; 0.20–0.29 = Marginal; and <0.20 = Poor. The test–retest reliability was assessed using intraclass correlation coefficient (ICC) in 35 respondents. The first and second test data collections were conducted within 2 weeks on respondents who matched the inclusion criteria. Good reliability was indicated by ICC values between 0.6 and 0.8, while values above 0.8 were considered optimal. Floor and ceiling effects were also tested in the OFI-8-ID to analyze the proportion of lowest and highest scores. The collected data were processed and analyzed using SPSS version 27.0, IBM Corp, NY, USA, 2020.

## 3 Results

### 3.1 Translation and cross-cultural adaptation

The forward-translation involved two independent translators fluent in English translating the OFI-8 from English into Indonesian. The first translator (T1) had a medical background, while the second translator (T2) was a non-medical translator. Both translators were from the TransMedical Institute in Jakarta. There were no significant differences observed in the translations provided by T1 and T2. The main difference was that T1 used the word “gigi tiruan” for the word “denture” in Question 3, whereas T2 used the word “gigi palsu.” Another difference was found in Question 7, where T1 used the word “menyikat” for the word “brush,” whereas T2 used the word “menggosok.” The translation results were then synthesized by a committee of experts consisting of a clinical prosthodontist, a clinical epidemiologist, and an expert gerodontist in order to achieve consensus and generate a T12 translation. The expert committee agreed to use more common, appropriate, and easily understandable words since the respondents were older adults. The results of the synthesis can be seen in Supplementary Table 1.

The T12 synthesis results were subsequently back-translated into English by two translators who did not have a medical background and were unaware of the concept of the OFI-8 instruments. They were native English speakers who were also fluent in Indonesian. The two translators were from the TransMedical Institute in New Zealand (BT1) and Australia (BT2). The expert committee assessed both back-translation results to ascertain whether they accurately represented the original language and found no discrepancies in meaning. In this step, the expert committee members also discussed the type of hard food mentioned in Question 6. The original version of the OFI-8 mentions “squid jerky or pickled radish” as an example of hard food. In Indonesia, those foods are rarely found or consumed. Therefore, the expert committee agreed to replace the food with “beef jerky” and “pickled cucumbers or carrots.” The expert committee discussed the results until final consensus was reached. The Indonesian version of the OFI-8 is shown in Table 2.

**Table 2:** The Indonesian version of the OFI-8 (OFI-8-ID).

No	English version of OFI-8^a^	OFI-8-ID
1.	Do you have any difficulties eating tough food compared to 6 months ago?	Apakah saat ini Anda mengalami kesulitan makan makanan keras bila dibandingkan dengan 6 bulan yang lalu?
2.	Have you choked on your tea or soup recently?	Apakah Anda pernah tersedak saat minum teh atau makan sup baru-baru ini?
3.	Do you use dentures?^a^	Apakah Anda menggunakan gigi palsu?^a^
4.	Do you often have a dry mouth?	Apakah Anda sering merasa mulut Anda kering?
5.	Do you go out less frequently than you did last year?	Apakah Anda lebih jarang bepergian keluar rumah bila dibandingkan dengan tahun lalu?
6.	Can you eat hard foods like squid jerky or pickled radish?	Apakah Anda bisa makan makanan keras seperti dendeng daging sapi atau acar mentimun/wortel?
7.	How many times do you brush your teeth in a day? (two or more times/day)	Berapa kali Anda menggosok gigi dalam sehari? (2 kali/hari atau lebih)
8.	Do you visit a dental clinic at least annually?	Apakah Anda mengunjungi klinik gigi setidaknya sekali dalam setahun?

a Jika Anda kehilangan satu atau lebih gigi, penting untuk menggunakan gigi palsu agar Anda tetap dapat mengunyah makanan yang keras.

### 3.2 Respondents characteristics

Sociodemographic and health characteristics of the 145 respondents are summarized in Table 3. The average age of respondents who completed the OFI-8-ID was 68 years (SD, 5.7; range 60–86). Female respondents (*n* = 105, 72.4%) outnumbered male respondents. There were 76 respondents with hypertension and 17 respondents with random blood sugar levels ≥200 mg/dL. Clinical oral conditions of respondents are presented in Table 4. Tooth loss was observed in all respondents, with 21 being fully edentulous; nevertheless, only 40 respondents used dentures.

**Table 3:** Overview of sociodemographic and health characteristics of the participants.

Characteristic	Mean (SD)	*n* (%)
Age	68 (5.7)	
Gender		
Male		40 (27.6)
Female		105 (72.4)
Education level		
Primary		46 (31.7)
Secondary		64 (44.1)
Tertiary		35 (24.1)
Hypertension		
No		69 (47.6)
Yes		76 (52.4)
Blood sugar test (random)		
<200 mg/dL		128 (88.3)
≥200 mg/dL		17 (11.7)

**Table 4:** Clinical characteristics of the participants.

Characteristic	Mean (SD)	*n* (%)
Missing teeth	10 (4.6)	
Fully edentulous		21 (14.5)
Partially edentulous		124 (85.5)
Denture use		
No denture		105 (72.4)
Partial denture		31 (21.4)
Full denture		9 (6.2)
OFI-8-ID score	4.30 (2.3)	

### 3.3 Responses to the OFI-8-ID

Respondent responses to each OFI-8-ID item are shown in Table 5. Overall, 51.7% of respondents experienced difficulty in eating tough foods compared to 6 months earlier, and 69.7% of respondents had choked on tea or soup, indicating a swallowing problem. There were no missing data, and the mean OFI-8-ID score was 4.30 (SD, 2.3).

**Table 5:** Distribution response items of the OFI-8 (item-level performance).

Oral Frailty Index-8	Yes, *n* (%)	No, *n* (%)
Do you have difficulties eating tough foods compared to 6 months ago?	75 (51.7)	70 (48.3)
Have you choked on your tea or soup recently?	101 (69.7)	44 (30.3)
Do you use denture?	40 (27.6)	105 (72.4)
Do you often have a dry mouth?	62 (42.8)	83 (57.2)
Do you go out less frequently than you did last year?	73 (49.7)	72 (50.3)
Can you eat hard foods like squid jerky or pickled cucumber/carrot?	47 (32.4)	98 (67.6)
How many times do you brush your teeth in a day? (two or more times/day)	117 (80.7)	28 (19.3)
Do you visit a dental clinic at least annually?	37 (25.5)	108 (74.5)

### 3.4 Validity and reliability of the OFI-8-ID

The I-CVI values ranged from 0.67 to 1, and the S-CVI/Ave was 0.95, indicating good content validity of the OFI-8-ID. The results of content validity assessment are presented in Supplementary Table 2.

The OFI-8-ID was subsequently tested on 33 respondents to evaluate face validity qualitatively and quantitatively. The characteristics of the respondents are shown in Supplementary Table 3. All respondents completed the prefinal version of the OFI-8-ID through a direct interview. Respondents were asked to answer the questions based on their situation using the available answer choices and to provide feedback on any words or sentences that were difficult to understand or unclear. A total of 31 respondents were able to understand the questions and answer the, easily, only two respondents needed to read Question 7 twice because they were confused about the brushing frequency options listed. Quantitatively, the I-FVI values ranged from 0.83 to 1 and the S-FVI/Ave was 0.93, indicating good face validity of the OFI-8-ID.

Validity test results for the OFI-8-ID are listed in Table 6. The OFI-8-ID demonstrated excellent validity, as seen by positive Pearson’s correlations, *p*-values below 0.05, and *r*-count values for all eight items exceeding *r-*table values (the *r-*table value for a sample size of 145 is 0.163). In order to determine concurrent validity, the total score of the OFI-8-ID was correlated with the total number of missing teeth, demonstrating a strong positive correlation (*r* = 0.526), signifying that increased tooth loss correlates with a higher OFI score*.* This finding shows that the OFI-8-ID has good concurrent validity.

**Table 6:** Validity test results of the OFI-8-ID.

OFI-8-ID	*r*	*p*-Value
Q1	0.74	<0.001*
Q2	0.65	
Q3	0.71	
Q4	0.73	
Q5	0.52	
Q6	0.69	
Q7	0.86	<0.002*
Q8	0.68	<0.004*

* Pearson's correlation coefficient (*p* < 0.05).

The KMO value for the OFI-8-ID was 0.497, which was slightly below the commonly accepted minimum threshold of 0.50, while Bartlett's test of sphericity was statistically significant [*χ*^2^(28) = 76.322, *p* ≤ 0.001]. Principal component analysis suggested four components. However, the marginal KMO value indicated that data were not optimally suited for stable factor extraction. Sensitivity analyses excluding Q5 and subsequently excluding Q2 slightly improved the KMO values to 0.507 and 0.538, respectively, with statistically significant Bartlett's tests.

Internal consistency results for the OFI-8-ID are shown in Table 7. The OFI-8-ID demonstrated moderate internal consistency, with a Cronbach's alpha of 0.65. The corrected item-total correlations ranged from 0.15 to 0.61. Items Q1, Q3, Q4, Q6, Q7, and Q8 demonstrated satisfactory correlations with the total score, whereas Q2 and Q5 showed lower correlations. The ICC for test–retest reliability of total score OFI-8-ID was 0.92 and varied from 0.68 to 1 for eight questions of OFI-8-ID. The findings indicated that the OFI-8-ID instrument was reliable. The outcome details are provided in Table 8.

**Table 7:** Summary of reliability assessment.

Item-level reliability		
Item number	Item label	Item-total correlation
Q1	Eating difficulty	0.61
Q2	Swallowing difficulty	0.31
Q3	Denture use	0.38
Q4	Dry mouth	0.35
Q5	Social participation	0.15
Q6	Eating hard food	0.58
Q7	Brushing teeth	0.34
Q8	Dental visit	0.38
Scale-level reliability		
Internal consistency	Cronbach's alpha	0.65
Test–retest reliability	Intraclass correlation	0.92

**Table 8:** Test–retest reliability of the OFI-8-ID using intraclass correlation coefficient (ICC).

OFI-8-ID	ICC	95% CI		*p*-Value
		Min.	Max.	
Q1	0.84	0.71	0.91	<0.001
Q2	0.94	0.89	0.97	
Q3	1	—	—	0.000
Q4	0.94	0.89	0.97	<0.001
Q5	0.94	0.89	0.97	
Q6	0.83	0.69	0.91	
Q7	0.91	0.84	0.95	
Q8	0.68	0.46	0.82	
Total score OFI-8-ID	0.92	0.79	0.96	

Floor and ceiling effect analysis was performed on the OFI-8-ID, revealing no significant effects. The proportion of respondents scoring the minimum possible OFI-8 score (scorep = 1) was 8.3% (*n* = 12), while only 3.4% (*n* = 5) achieved the maximum possible score (score = 11). Both values were well below the commonly accepted 15% threshold (Supplementary Table 4).

## 4 Discussion

Aging in the older adults is accompanied by a decline in various bodily functions, including oral function. There are several valid and reliable instruments for assessing the risk of oral frailty, such as the OF-5, SOFT, OMFI, and OFI-8. In this study, we selected the OFI-8 because it is more applicable in community settings since it does not need any specialized equipment, whereas OF-5 still need instruments (oral diadochokinesis) to assess articulatory oral motor skills. Meanwhile, SOFT and OMFI are still limited to preliminary studies with questionable validity and reliability (17). Furthermore, the OFI-8 also assessed oral health-related behavior and social participation, both of which are linked to oral function. The OFI-8 was unavailable in Indonesian; therefore, in order to use it, cross-cultural adaptation, validity assessment, and reliability evaluation were necessary. Each component of a measurement instrument must be comprehensible to its respondents, suitable for the intended respondents, and have a distinct objective. Moreover, it must be adapted to the language and culture of the targeted respondents. Consequently, item selection and determination must engage respondents through quantitative research.

Initially, the OFI-8 was translated from English to Indonesian by two translators, one with a medical and one from a non-medical background. Researchers decided to use *TransMedical* for the forward- and back-translation because this institution has translators from both medical and non-medical backgrounds who are experienced and trusted in translating medical documents. In addition to these two translators, a second team of native translators was also involved in the back-translation of the OFI-8 into English. These translators were fluent in Indonesian and also had the ability and mastery of English (their mother tongue) to back-translate the synthesized OFI-8. After the backward blind translations to English, the committee of researchers, translators, and experts conducted a thorough review of all discrepancies and semantic ambiguities until they reached a consensus. To accomplish semantic equivalence, ensure standardized terminology, and produce items with an understandable, clear, and accurate message, a variety of decisions were made. The following are a few examples:

1. The terms “gigi palsu,” which means dentures, and “menggosok gigi,” which means brushing teeth, were preferred over “gigi tiruan” and “menyikat gigi,” respectively, because they are more familiar and understandable to the respondents.
2. Types of hard foods were adapted to Indonesian culture, so the expert committee replaced squid jerky and pickled radish with beef jerky and pickled cucumber or carrot, respectively, since Indonesian people are more familiar with the latter two.
3. The original item specified the frequency of brushing teeth as three times or more; however, it was formally changed to two times or more.

The OFI-8-ID demonstrated excellent content validity based on the CVI values. The high I-CVI and S-CVI values indicate strong agreement among experts regarding the relevance of the translated items to the construct of oral frailty. These findings confirm that the translation and cultural adaptation process preserved the conceptual meaning of the original instrument while ensuring its appropriateness for the Indonesian cultural and linguistic context. Face validity was evaluated among older adults, who reported that the questionnaire was clear, understandable, and easy to complete. The high FVI indicates that the translated items were well accepted by the target population and interpreted as intended. Minor modifications were made following expert review to improve clarity and cultural appropriateness without altering the conceptual meaning of the original items. Such revisions are consistent with recommendations for cross-cultural adaptation, which emphasize conceptual rather than literal equivalence. This finding suggests that the Indonesian OFI-8 is suitable for self-administration among community-dwelling older adults.

In this present study, we observed tooth loss in all respondents, but only 40 reported using dentures. Several studies have shown that individuals with 20 or more teeth are less susceptible to dementia and frailty, underscoring the importance of replacing missing teeth with dentures (26–29). This initial study investigated and confirmed that the OFI-8-ID possesses good validity and reliability for assessing risk of oral frailty in Indonesian older adults. The concurrent validity of the OFI-8-ID was evaluated by examining the correlation between the total OFI-8-ID score and the number of missing teeth, a criterion selected to determine the extent to which the two theoretically related measures were associated. The findings indicated that an increase in tooth loss correlates positively with a higher OFI-8-ID scores, which indicate higher risk of oral frailty. This outcome is consistent with numerous previous studies. A meta-analysis by Zhang et al. reported a significant negative correlation between the number of teeth and frailty. In particular, individuals with fewer than 20 teeth exhibited a higher risk of frailty compared with those with more than 20 teeth (30). Individuals with fewer than 20 remaining teeth also tend to experience masticatory dysfunction. A systematic review by Kojima et al. addressed the association between masticatory dysfunction and frailty. Their review found that older individuals with masticatory dysfunction had approximately a 1.83-fold increased risk of frailty compared with those with normal masticatory function, emphasizing the importance of masticatory ability. Our findings corroborate this, as tooth count directly influences masticatory function. Thus, a reduced number of teeth can elevate the risk of frailty among older adults, aligning with Kojima's conclusions (31).

The use of missing teeth as an external criterion should be interpreted with caution. Tooth loss is clinically relevant to oral frailty because it may affect masticatory function, dietary intake, denture needs, and functional oral decline. A meta-analysis by Paul et al. stated that there is sufficient evidence to consider tooth loss an important indicator for the development of frailty (32). Retaining at least 20 functional teeth is essential for effective chewing, digestion, and nutritional intake, all of which directly influence overall health and contribute to frailty prevention (32). Identifying tooth count as a practical clinical marker enables targeted interventions for frailty prevention. Nevertheless, oral frailty is a multidimensional condition that cannot be fully represented by missing teeth alone. The OFI-8 includes broader dimensions, such as chewing and swallowing difficulties, dry mouth, oral health behaviors (denture use, dental attendance, tooth-brushing behavior) and social participation. Consequently, the observed correlation between OFI-8-ID scores and the number of missing teeth demonstrates concurrent validity; however, it remains insufficient for establishing diagnostic validity in the absence of a gold-standard comparison.

The EFA provided limited evidence for construct validity. The initial KMO value for eight OFI-8-ID items was slightly below the commonly accepted minimum threshold, although Bartlett's test of sphericity was statistically significant. This suggests that while correlations existed among items, the sample and item correlation patterns were not optimal for stable factor extraction. THE OFI-8 covers several clinically distinct domains, including chewing difficulty, swallowing difficulty, denture use, oral dryness, social participation, ability to eat hard food, tooth-brushing frequency, and dental visits. Therefore, a single-factor structure is not necessarily expected. Sensitivity analyses excluding Q5 and subsequently excluding both Q2 and Q5 slightly improved the KMO values, with statistically significant Bartlett's tests. However, deleting these items would alter the original structure and content coverage of the OFI-8, particularly because Q2 represents swallowing difficulty and Q5 represents social participation, which are theoretically and clinically important to the construct of oral frailty. Therefore, we retained the original eight-item structure of the OFI-8-ID and did not modify the instrument solely on the basis of the EFA results.

The internal consistency of the OFI-8 was 0.65, falling below the recommended threshold of ≥0.70. However, deletion of any individual item resulted in only minimal changes in Cronbach's alpha, indicating that no single item substantially affected the internal consistency of the instrument. These findings support retaining all eight items in the Indonesian version of the OFI-8. This value was interpreted cautiously because the OFI-8 is a brief multidimensional screening index that includes oral function, oral health behavior, denture use, dental attendance, and social participation rather than a single homogenous construct. The reliability of the OFI-8-ID was evaluated using test–retest analysis. The results showed that this instrument is reliable and consistent for use. Only one item achieved a maximal value of 1, because the question was about whether the respondents use dentures or not. The lowest value was observed for social participation, though it had good intraclass coefficient value.

Floor and ceiling effect analysis revealed no significant effects in this instrument. If a questionnaire has a floor and ceiling effect, it is relatively less sensitive and cannot precisely measure the minimum and maximum scores of the sample. The absence of significant floor and ceiling effects suggests that the OFI-8-ID has an adequate measurement range to assess oral frailty across the target population.

By enhancing the usability of the OFI-8-ID, healthcare professionals can potentially enhance the quality of life of older individuals by detecting, intervening, and monitoring their oral health through preventive care. Moreover, incorporating oral frailty assessment into routine geriatric assessments can facilitate comprehensive care planning and interdisciplinary collaboration among health professionals. With the introduction of the OFI-8-ID, health professionals and researchers will have a valuable tool for assessing oral frailty in older adults in Indonesia. This validated and standardized questionnaire will facilitate the early detection of oral frailty, thereby enabling targeted intervention and preventive strategies. Given the current aging demographic, comprehending and addressing oral frailty are crucial for promoting healthy aging and enhancing overall wellbeing. By identifying individuals at risk, the OFI-8-ID can facilitate the provision of appropriate oral health services, ultimately contributing to improved oral health outcomes and quality of life.

The OFI-8-ID is a valuable tool for evaluating oral frailty in older adults in Indonesia due to its numerous advantages. Its reliability and consistency are guaranteed by its standardized and validated nature, while its simplicity in administration and scoring makes it easy to integrate into routine assessments. The implementation of OFI-8-ID also enables the collection of data on oral frailty in the Indonesian older adult population, thereby enhancing comprehension of its prevalence and influence on health outcomes.

The current study provides a valuable perspective on the psychometric validity of the OFI-8-ID questionnaire among the older adult population of Indonesia. However, there are several limitations worth discussing. First, the sample size was relatively small and the respondents were recruited from only one location, which may limit the generalizability of findings to larger populations. Second, concurrent validity was assessed using the number of missing teeth as an external clinical criterion rather than a comprehensive oral frailty reference standard. Although tooth loss is clinically relevant, it does not capture all dimensions of oral frailty. In order to reinforce the validity of the findings and address these limitations, it is advisable to conduct additional research with a more diverse and expansive population. Further validation studies should also compare the OFI-8-ID with comprehensive clinical oral function assessments and validated Oral Health-Related Quality-of-Life (OHRQoL) instruments.

## 5 Conclusion

The OFI-8-ID demonstrated good psychometric qualities and is a valid and reliable tool for measuring oral frailty in Indonesia's older adult population.

## Data Availability

The datasets presented in this article are not readily available. Data may be shared upon reasonable request to the corresponding author. Requests to access the datasets should be directed to astari.dds@gmail.com.
