# Supplementary material for: Cross-cultural adaptation and psychometric validation of the simplified Oral Frailty Index (OFI-8) among Indonesian older adults
Source: Front Oral Health. 2026 Aug 7;7:1853252. doi: 10.3389/froh.2026.1853252 (PMC13493268; doi:10.3389/froh.2026.1853252)
Supplement: Supplementary file 1 [file Table1.docx]

Supplementary Material

# 1 Supplementary Table

**Supplementary table 1.** Synthesize result of OFI-8 T12 in Bahasa Indonesia

| No | English Version of OFI-8 | OFI-8 T12 |
| --- | --- | --- |
| 1. | Do you have any difficulties eating tough food compared to 6 months ago? | Apakah saat ini Anda mengalami kesulitan makan makanan keras bila dibandingkan dengan 6 bulan yang lalu? |
| 2. | Have you choked on your tea or soup recently? | Apakah Anda pernah tersedak saat minum teh atau makan sup baru-baru ini? |
| 3. | Do you use dentures? | Apakah Anda menggunakan gigi palsu? |
| 4. | Do you often have a dry mouth? | Apakah Anda sering merasa mulut Anda kering? |
| 5. | Do you go out less frequently than you did last year? | Apakah Anda lebih jarang bepergian bila dibandingkan dengan tahun lalu? |
| 6. | Can you eat hard foods like squid jerky or pickled radish? | Apakah Anda bisa makan makanan keras seperti dendeng cumi atau acar lobak? |
| 7. | How many times do you brush your teeth in a day? (2 or more times/day) | Berapa kali Anda menggosok gigi dalam sehari? (2 kali/hari atau lebih) |
| 8. | Do you visit a dental clinic at least annually? | Apakah Anda mengunjungi klinik gigi setidaknya sekali dalam setahun? |
|  | ^a^ If you have lost a tooth, it is important to treat it with a denture so that you can eat hard food | ^a^ Jika Anda kehilangan gigi, penting untuk mengatasinya dengan pembuatan gigi palsu agar Anda dapat mengonsumsi makanan yang keras. |

**Supplementary table 2.** Content Validity Assessment

| OFI-8-ID | Expert 1 | Expert 2 | Expert 3 | I-CVI | S-CVI/Ave |
| --- | --- | --- | --- | --- | --- |
| Q1 | 4 | 4 | 3 | 1 | 0.95 |
| Q2 | 4 | 4 | 4 | 1 |  |
| Q3 | 4 | 3 | 4 | 1 |  |
| Q4 | 3 | 2 | 4 | 0.67 |  |
| Q5 | 4 | 3 | 3 | 1 |  |
| Q6 | 4 | 4 | 4 | 1 |  |
| Q7 | 4 | 4 | 4 | 1 |  |
| Q8 | 3 | 4 | 3 | 1 |  |

(1 = not relevant; 2 = somewhat relevant; 3 = quite relevant; 4 = highly relevant)

**Supplementary table 3.** Characteristics of participants in OFI-8-ID pre-test

| Characteristic | OFI-8-ID Pre test  (n=33) |
| --- | --- |
| Age   60-70   >70 | 17 (51.5%)  16 (48.5%) |
| Gender  Male  Female | 16 (48.5%)  17 (51.5%) |
| Education level  Secondary  Tertiary | 9 (27.3%)  24 (72.7%) |
| Completion time | 3-5 minutes |

**Supplementary table 4**. Floor and ceiling effect result of OFI-8-ID

|  | ***Floor effect*** | | ***Ceiling effect*** | |
| --- | --- | --- | --- | --- |
|  | **n** | **%** | **n** | **%** |
| OFI-8-ID (n=145) | 12 | 8.3% | 5 | 3.4% |
